# Functional characterization of the vaccinia virus I5 protein

**DOI:** 10.1186/1743-422X-5-148

**Published:** 2008-12-15

**Authors:** Bethany Unger, R Jeremy Nichols, Eleni S Stanitsa, Paula Traktman

**Affiliations:** 1 Department of Microbiology & Molecular Genetics, Medical College of Wisconsin, Milwaukee, WI 53226, USA; 2 MRC Protein Phosphorylation Unit, Univ. of Dundee, Dundee, UK; 3 McArdle Laboratory for Cancer Research, Univ. of Wisconsin, Madison, WI, USA

## Abstract

The I5L gene is one of ~90 genes that are conserved throughout the chordopoxvirus family, and hence are presumed to play vital roles in the poxvirus life cycle. Previous work had indicated that the VP13 protein, a component of the virion membrane, was encoded by the I5L gene, but no additional studies had been reported. Using a recombinant virus that encodes an I5 protein fused to a V5 epitope tag at the endogenous locus (vI5V5), we show here that the I5 protein is expressed as a post-replicative gene and that the ~9 kDa protein does not appear to be phosphorylated *in vivo*. I5 does not appear to traffic to any cellular organelle, but ultrastructural and biochemical analyses indicate that I5 is associated with the membranous components of assembling and mature virions. Intact virions can be labeled with anti-V5 antibody as assessed by immunoelectron microscopy, indicating that the C' terminus of the protein is exposed on the virion surface. Using a recombinant virus which encodes only a TET-regulated copy of the I5V5 gene (vΔ*ind*I5V5), or one in which the I5 locus has been deleted (vΔI5), we also show that I5 is dispensable for replication in tissue culture. Neither plaque size nor the viral yield produced in BSC40 cells or primary human fibroblasts are affected by the absence of I5 expression.

## Background

Vaccinia virus, the prototypic poxvirus, replicates solely in the cytoplasm of infected cells. This physical autonomy is accompanied by genetic autonomy: the 192 kb DNA genome, encodes ~200 proteins involved in diverse aspects of the viral life cycle [1]. A virally encoded transcriptional apparatus directs three temporally regulated phases of gene expression, and a virally encoded replication apparatus mediates genome replication and maturation. A large number of proteins contribute to the complex process of morphogenesis, which culminates in the production of mature virions (MV) [2]. Most MV remain within the cell, but a subset becomes enwrapped in two extra membranes derived from the Golgi apparatus or late endosomal compartment; these wrapped virions are then released by exocytosis as enveloped virions (EV) and mediate cell-to-cell and distal spread [3,4]. Finally, a significant number of the viral genes encode proteins that interface with the host. Some of these proteins regulate intrinsic cellular responses to infection such as apoptosis and the antiviral response, whereas others represent extracellular mediators that interface with cytokines and cells of the immune system [1,5-10].

Comparison of the genomes of a large number of orthopoxviruses has led to the identification of ~90 genes that are fully conserved [11]. These genes are therefore thought to encode the repertoire of proteins required for the poxviral life cycle. A combination of genetic, cell biological and biochemical approaches have enabled the functional characterization of most, but not all, of these genes. One of the gene products that had not been studied in depth was the product of the I5L gene, which encodes a structural protein first identified as VP13 [12]. I5 is one of ~75 structural proteins identified by proteomic analyses as localizing to either the membrane or core of the mature poxvirus virion [2,13-15]. Core proteins include structural proteins essential for the assembly of the virion core, the full complement of proteins required for mediating the early phase of gene expression, and virally encoded kinases and phosphatases. The MV membrane contains ~20 proteins, many of which contribute to virion morphogenesis [2]. At least 11 membrane proteins are essential for virion entry [16-19], and others mediate the association of virions with GAGs or laminins on the cell surface [20-24]. Other membrane proteins appear to be dispensable in vitro but contribute to pathogenesis in vivo [25]. Because our laboratory has a long-standing interest in virion morphogenesis and in the function of virion membrane proteins, we undertook an analysis of the I5 protein.

## Methods

### Materials, cells and viruses

African green monkey kidney BSC40 cells and human TK^- ^143B cells were cultured in Dulbecco's modified Eagle medium (DMEM) containing 5% fetal calf serum (FCS, Invitrogen, Carlsbad, CA) at 37°C in the presence of 5% CO_2_; human diploid fibroblasts (kindly supplied by S. Terhune, Medical College of Wisconsin, Milwaukee, WI) were cultured similarly except that the medium contained 10% FCS. Viral stocks (WR strain of vaccinia virus) were prepared by ultracentrifugation of cytoplasmic lysates through 36% sucrose; titration was performed on confluent monolayers of BSC40 cells, which were fixed and stained with 0.1% crystal violet in 3.7% formaldehyde at 48 hpi. Restriction endonucleases, T4 DNA ligase, calf intestinal alkaline phosphatase (CIP) and Taq polymerase were purchased from Roche Applied Sciences (Indianapolis, IN). Geneticin (G418 sulfate), Lipofectamine 2000, monoclonal V5 antibody, protein molecular weight markers and DNA molecular weight standards were purchased from Invitrogen (Carlsbad, CA). ^32^P-orthophosphate and ^35^S-methionine were purchased from Perkin-Elmer Life and Analytical Sciences, Inc. (Boston, Mass.). Ultra pure chemicals, Protein A sepharose and Protein G agarose were from Sigma Aldrich (St. Louis, MO). DNA oligonucleotides were synthesized by IDT (Coralville, Iowa).

### Construction of recombinant viruses

Recombinant viruses were prepared as described below, using the primers described in Table 1.

**Table 1:** 

Virus	Primer Name	Primer Sequence *
I5V5		

	PN 5'	5' AT**GGATCC**GGAAGGGTATCTATACTTATAG 3'

	I5V5 3'	5' TCCCAACAAAGGGTTAGGGATAGGTTTACCACT 3'

	I5V5 5'	5' CCTAACCCTTTGTTGGGACTCGACAGTACTTAA 3'

	PN 3'	5' AT**GGATCC**GTTGAATAAATCCTCCATC 3'

	SCRN 5'	5' GTACGCTACGTACGTCAAATCCC 3'

	SCRN 3'	5' GGCATAATCCGGATGTTGTGTAG 3'

VΔ*ind*I5V5		

	1	5' GG**GGATCC***AAGCTT*CTAGGACTTTGTCAC 3'

	B	5' CTCTATCACTGATAGGGATATTTATATCTAAAAATTAGATC 3'

	C	5'CCCTATCAGTGATAGAGAATGGTGGATGCTATAAC 3'

	3'	5'AT**GGATCC**atcgatTTAAGTACTGTCGAGTCCCAACAAAGG GTTAGGGATAGGTTTACCACTTTTCATTAATAGGG 3'

vΔI5		

	I4 pUCneo Asp	5' GCGCCC*ggtacc*GAATAAATCCTCCATC 3'

	I4 pUCneo Bam	5' CG**GGATCC**GTACGTAAAATCCCTATT 3'

	I6pUCneo Bam/Hind	5' CG**GGATCC***AAGCTT*CTAAAAATTAGATCAAAG 3'

	I6 pUCneo Xba	5' ATTCTAGAGGCGGTGTGGATTTC 3'

*The restriction enzyme sites encoded in the primers are noted as follows: BamHI, bold; HindIII, italics; ClaI, lower case; Asp718, lowercase italics; XbaI, small caps.

### Generation of the vI5V5 virus

#### A) Cloning

The overlapping products of two initial PCR reactions (1: primers PN 5'+ I5V5 3'; 2: primers I5V5 5'+ PN3') were used together as the template for a second round of PCR performed with primers PN 5' and PN 3'. The final product was digested with Bam HI and cloned into pUCNEO [26], forming pUCneo:I4-I5V5-I6. *B) Isolation of the virus by transient dominant selection with G418*. Cells were infected with wt virus and transfected with pUCneo: I4-I5V5-I6; at 15 hpi G418 was added to select for viruses in which the plasmid had been inserted into the viral genome. Cells were harvested at 48 hpi and two rounds of plaque purification were performed to purify G418^R ^viruses; insertion of the plasmid was confirmed by PCR with primers specific for NEO. Two sequential rounds of plaque purification in the absence of G418 were then performed to allow recombinational resolution of the tandem repeats present in this virus. To distinguish viruses containing only the wild-type allele from those containing the V5-tagged locus, plaque isolates were screened by PCR using primers that flank the I4 3' and I5 5' junction (I5 SCRN 5' or 3').

### Generation of the vΔindI5V5 virus

#### A) Cloning

The overlapping products of two initial PCR reactions (1: primers 1 +B; 2: primers C +3') were used together as the template for a second round of PCR performed with primers 1+3'; this product was digested with *Hind*III and *Cla I *and cloned into pJS4-tetR (final product pJS4:tetR ↔ opI5) [27]. The final plasmid contains two transcriptional cassettes. One drives constitutive expression of the TET repressor (tetR), and the other contains the V5I5 ORF under the regulation of the TET operator and the I5 promoter. These two cassettes are flanked by the left and right halves of the TK gene, which enables insertion into the genome by homologous recombination. *B) Isolation of the virus by BrdU and G418 selection*. Cells were infected with wt virus and transfected with linearized pJS4:tetR ↔ opI5 DNA. TK^- ^virus was isolated by two rounds of plaque purification on human TK^- ^cells in the presence of BrdU (25 μg/ml). Plaques of the correct genotype were expanded. To generate the final inducible virus, the endogenous I5 allele was then replaced with a NEO cassette using the I5 KO plasmid as described below.

### Generation of the vΔI5 virus

#### A) Cloning: generation of the I5KO plasmid

Two PCR reactions were performed, one with primers I4 pUCneo *Asp *+ I4 pUCneo *Bam*, and one with primers I6pUCneo *Bam/Hind *+ I6 pUCneo *Xba*. The products were digested with *Asp*718 + *BamH*I and *BamH*I + *Xba*I, respectively, and then ligated simultaneously into pBSIIKS plasmid DNA that had been digested with *Asp*718 and *Xba*I. The resultant plasmid, pBSIIKS:I4-I6, was used as the recipient in the next round of cloning. A 1.3 kb fragment containing the NEO gene under the regulation of a constitutive viral promoter was released from pUCneo by digestion with *Bam*HI and *Hind*III, and the 5' overhangs were filled in with the Klenow fragment of *E. coli *DNA polymerase. The plasmid pBSIIKS:I4-I6 was linearized at the internal BamHI site and treated with calf intestinal alkaline phosphatase; the 5' overhangs were filled in as described above. This linearized plasmid was then ligated with the NEO insert; the resulting plasmid, in which portions of the I4 and I6 gene flank NEO, was designated pI5KO. *B) Isolation of the virus by G418 selection*. Cells were infected with vI5V5 and transfected with pI5KO; G418^R ^virus was obtained by two rounds of plaque purification in the presence of G418; correct insertion of the NEO cassette and deletion of the I5 locus were confirmed by PCR.

### Metabolic labeling and immunoprecipitation

Confluent monolayers of BSC40 cells were infected with vI5V5 or wt virus (MOI 5) and metabolically labeled with ^35^S-methionine (100 μCi/ml) from 3–9 hpi or with ^32^PPi (100 μCi/ml) from 4–8 hpi in DMEM lacking methionine or phosphate, respectively, along with 5% FCS that had been dialyzed against TBS. Cells were lysed in phospholysis buffer (10 mM NaPO_4 _[pH 7.4], 100 mM NaCl, 1% Triton X-100, 0.1% SDS, 0.5% sodium deoxycholate), and clarified lysates were incubated with either monoclonal V5 or polyclonal F18 antisera for 4 hr on ice followed by the addition of Protein A for 1.5 hours. Immune complexes were washed, resolved electrophoretically, and visualized by autoradiography using a Kodak low emission screen.

### Immunoelectron microscopy

#### Infected cells

BSC40 cells were infected with vI5V5 (MOI 2) for 17 hr and then fixed in situ with 4% paraformaldahyde/0.1% glutareldahyde in PBS for 60 min at room temp. Cells were then processed for immunoelectron microscopy and embedded in Lowicryl K4M; grids were stained using the V5 antibody followed by incubation with a secondary antibody conjugated to 5 or 10 nm gold.*Intact virions: *Purified vI5V5 virions (or control virions encoding a wt I5 protein lacking the V5 epitope) were applied to grids and probed with either a control antibody (anti-A17) or the anti-V5 antibody and a secondary antibody conjugated to 10 nm gold particles. Grids were post-stained with uranyl acetate. All images were obtained on a Hitachi H-600 microscope.

### Immunofluorescence microscopy

BSC40 cells were infected with wt virus or vI5V5 for 8 h prior to being fixed with 4% paraformaldehyde; cells were then incubated with the monoclonal anti-V5 antibody and a secondary antibody conjugated to Alexafluor 594. DAPI was included to visualize the nucleus and viral replication factories.

### NP40 and DTT fractionation

wt and I5V5 virions purified by sedimentation on 25–40% sucrose gradients were treated with NP40 (1%) or NP40 and DTT (1% and 50 mM, respectively) [5]. The soluble (S) and particulate (P) fractions, representing the membrane and core components, respectively, were resolved by sedimentation (16,000 × g, 30 min, room temperature) and analyzed by immunoblot analysis with anti-V5 and antibodies against known membrane (A17) and core (F18) proteins.

### Protease treatment

I5V5 virions (6 μg) were subjected to treatment with chymotrypsin (Chymo) or trypsin (Tryp) (10 μg/ml, 30 min at 37°C) or with proteinase K (ProtK; 50 μg/ml) for 10 or 30 min at 4°C. After sedimentation at 14,000 × g, 5 min, the soluble (S) and pellet (P) fractions were resolved and analyzed by immunoblot analysis using anti-D8 or anti-V5 antibodies.

## Results and discussion

### I5 is expressed as a post-replicative protein, does not appear to be phosphorylated, and is present in crescents, immature and mature virions

**Figure 1:**
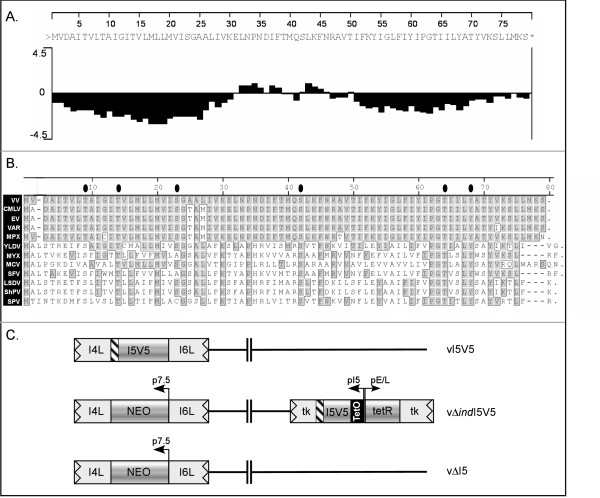
**Tools for characterization and analysis of the I5 protein**. (A) A Kyte-Doolittle hydrophilicity plot of the I5 protein was generated using the Protean module of the Lasergene software (DNASTAR, Inc.). (B) An alignment of the I5 homologs encoded by several chordopoxviruses is shown; sequences were obtained from  and aligned using the Megalign module of the Lasergene software. Vaccinia virus (VV, GenBank ID 29692238), camelpox virus (CMLV, GenBank ID 18640364), ectromelia virus (EV, GenBank ID 22164721), variola virus (VAR, GenBank ID 439035), monkeypox virus (MPV, GenBank ID 179750543), Yaba-like disease virus (YLDV, GenBank ID 12085087), myxoma virus (MYX, GenBank ID 18426922), molluscum contagiosum virus (MCV, GenBank ID 1492060), Shope fibroma virus (SFV, GenBank ID 18448493), lumpy skin disease virus (LSDV, GenBank ID 151505037), sheeppox virus (ShPV, GenBank ID 21492557), and swinepox virus (SPV, GenBank ID 18640187). (C) The key genomic features of three recombinant viruses used in this study are shown. In the vI5V5 virus, the endogenous locus has been modified such that the I5 protein is fused to a C-terminal V5 epitope tag. In the inducible vΔ*ind*I5V5 recombinant, the endogenous I5 ORF has been replaced by a NEO cassette, and the tetR gene and an inducible copy of the I5V5 ORF has been inserted into the non-essential thymidine kinase (TK) locus of the genome. The inducible I5V5 ORF is under the regulation of the I5 promoter and the TET operator. The vΔI5 virus was generated by replacing the I5V5 locus of vI5V5 with the NEO cassette; this virus is deleted for the I5 locus. The primers and strategy used for the generation of these viruses are shown in Table 1.

The I5L gene encodes a protein of 78 aa, which is predicted to have two highly hydrophobic domains at the N- and C-termini and is likely to be an integral membrane protein (Fig 1A). Indeed, VP13 was originally identified as a component of the membrane of mature virions [12]. The protein is highly conserved in diverse chordopoxviruses, as is evident in the alignment shown in Fig 1B. To enable a structural and functional characterization of I5, we generated a number of recombinant viruses, for which the key genomic features are depicted in Fig 1C. These recombinant viruses will be discussed in more detail throughout the remainder of this report.

**Figure 2:**
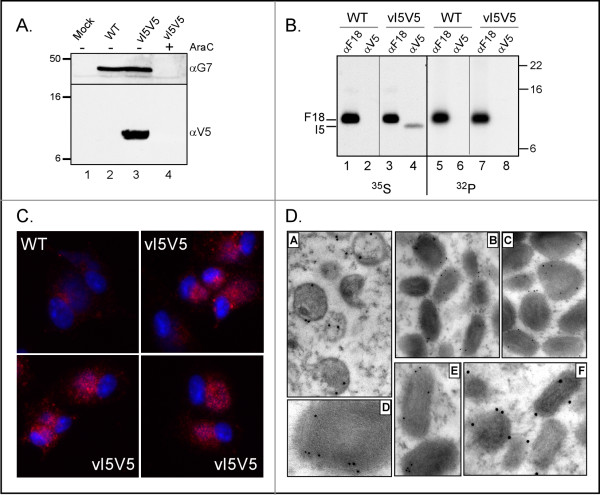
**Characterization of the endogenous I5V5 protein**. *(A). I5 is expressed as a late protein*. Cells were left uninfected (lane 1) or infected (MOI 5) with wt virus (lane 2) or with vI5V5 in the absence (lane 3) or presence (lane 4) of AraC (cytosine arabinoside, 20 μM). Cells were harvested at 8 hpi and lysates were probed with either an anti-G7 or anti-V5 antibody. The molecular masses (in kDa) of protein standards are shown at the left. *(B) I5 is not phosphorylated in vivo*. BSC40 cells were infected with wt virus (lanes 1,2,5,6) or vI5V5 (lanes 3,4,7,8) (MOI 5) in the presence of either ^35^S-met (lanes 1–4) or ^32^PPi (lanes 5–8) and then harvested for immunoprecipitation analysis. Immunoprecipitation was performed with antisera specific for the F18 protein (odd numbered lanes) or the V5 epitope (even numbered lanes); immune complexes were resolved electrophoretically and visualized by audioradiography. The molecular masses (in kDa) of protein standards are shown at the right. *(C) I5 shows a punctate distribution throughout the cytoplasm*. BSC40 cells were infected with wt virus or vI5V5 for 8 hpi; fixed cells were stained with the anti-V5 antibody and a secondary antibody conjugated to Alexafluor 594 and DAPI. *(D) I5 localizes to crescents, immature and mature virions*. Cells were infected with V5I5 (MOI 2) and harvested at 17 hpi for post-embedding immunoelectron microscopy. Sections were incubated with anti-V5 antibody and secondary antibodies conjugated to 5 nm (panels B, C, E) or 10 nm (panels A, D, F) gold particles.

First, we generated the vI5V5 virus, in which a V5 epitope tag has been inserted at the C-terminus of the endogenous I5 open reading frame (see Fig 1C). Immunoblot analysis permitted the ready visualization of the I5 protein in cells that had been infected with vI5V5, but not wild-type (WT) virus, for 8 h (Fig 2A). However, inclusion of ara C (cytosine arabinoside), which is an inhibitor of DNA replication and thus of intermediate and late gene expression, blocked the expression of I5. Hence, I5 is expressed as a post-replicative gene. The G7 protein, which is a known late protein, served as an internal control for the immunoblot analysis [28,29]. The predicted amino acid sequence of I5 contains a number of highly conserved serine, threonine and tyrosine residues that could be modified by phosphorylation *in vivo *(marked by black ovals in Fig 1B; ser/thr present in ≥ 10 of 13 orthologs shown). Therefore, we also performed immunoprecipitation analysis on infected cells that had been metabolically labeled with either ^35^S-met or ^32^PPi (Fig 2B). Although ^35^S-labeled I5 could be immunoprecipitated with the anti-V5 antibody, no ^32^P-labeled I5 was retrieved. In contrast, both ^35^S-labeled and ^32^P-labeled F18, a known phosphoprotein that is expressed at post-replicative times and encapsidated in the virion core, were retrieved in immunoprecipitations performed with the anti-F18 serum [30-33]. Thus, it appears that the I5 protein is not phosphorylated *in vivo*.

To monitor the intracellular localization of I5, we used immunofluorescence microscopy to visualize the I5 protein in cells that had been infected with vI5V5 for 8 h, or with wt virus as a negative control. DAPI was included to visualize nuclei and viral replication factories. As shown in Fig 2C, the I5V5 protein showed a punctate distribution that overlapped the viral replication factories and extended throughout the cytoplasm. Limited background staining was seen in cells infected with wild-type virus. The punctate staining was consistent with localization of I5 to intracellular membranes, although I5 was not restricted to any sub-cellular compartment such as the ER, the Golgi, or the plasma membrane.

Since the I5L gene encodes the protein identified as the VP13 component of purified virions, we utilized immunoelectron microscopy to determine whether I5 was associated with the classical intermediates in virion morphogenesis (crescents and immature virions) as well as with mature virions. As shown in Fig 2D, I5 was found to associate with crescents (A), immature (A) and mature virions (B-F).

### I5 is encapsidated in the virion membrane, and the C-terminus is exposed on the outer surface of intact virions

**Figure 3:**
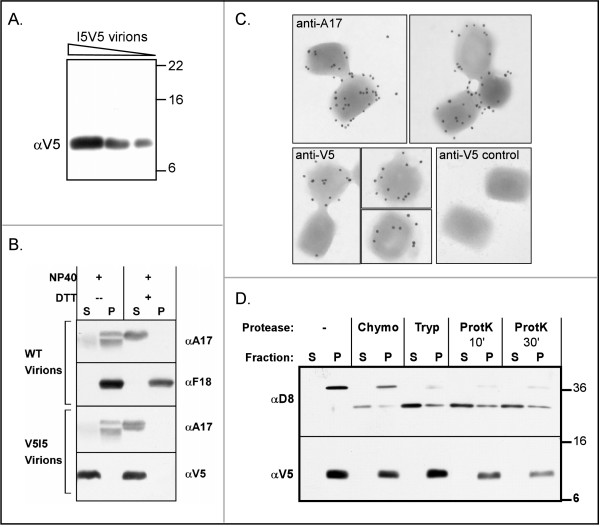
**Analysis of the I5 protein found within mature virions**. *(A) I5 is encapsidated within mature virions*. Increasing concentrations of purified I5V5 virions (0.5, 0.8, 2 μg) were subjected to immunoblot analysis and probed with the anti-V5 antibody. The 9 kDa I5V5 protein was readily visualized; the molecular masses (in kDa) of protein standards are shown at the right. *(B). I5 is found within the virion membrane*. wt and I5V5 virions purified by sedimentation on 25–40% sucrose gradients were treated with NP40 or NP40 and DTT (5). The soluble (S) and particulate (P) fractions, representing the membrane and core components, respectively, were resolved by sedimentation and analyzed by immunoblot analysis with anti-V5 and antibodies against known membrane (A17) and core (F18) proteins. *(C) The V5 epitope is accessible on the surface of intact I5V5 virions*. Purified vI5V5 virions (or a control virus encoding a wt I5 protein lacking the V5 epitope) were applied to grids and probed with either a control antibody (anti-A17) or the anti-V5 antibody and a secondary antibody conjugated to 10 nm gold particles. *(D). Proteolytic treatment of vI5V5 virions*. I5V5 virions (6 μg) were subjected to treatment with chymotrypsin (Chymo) or trypsin (Tryp) for 30 min or with proteinase K (ProtK) for 10 or 30 min. After sedimentation at 14,000 × g, 5 min, the soluble (S) and pellet (P) fractions were resolved and analyzed by immunoblot analysis using anti-D8 (top panel) or anti-V5 (lower panel) antibodies. The molecular masses (in kDa) of protein standards are shown at the right.

To assess the encapsidation of the I5 protein, purified vI5V5 virions were subjected to immunoblot analysis; as shown in Fig 3A, the I5V5 protein was readily observed. Virions were treated with NP40 or NP40+ DTT and then sedimented to prepare soluble (S) and pellet (P) fractions that contained membrane and core proteins, respectively [19,34]. The I5 protein was released into the soluble phase after treatment with NP40 alone (or NP40+DTT) (Fig 3B). We have previously observed that two other small membrane proteins, I2 and A13, behave similarly [19,34]. In contrast, the A17 protein, which is predicted to have two membrane spanning domains, contains intramolecular and intermolecular disulfide bonds, and binds to the dimeric A14 protein, is only released into the soluble phase upon treatment with both NP40 and DTT [28,35,36]. The F18 core protein remains in the pellet fraction after both treatments. Thus, the I5 protein is indeed a component of the virion membrane, consistent with the report that the I5L gene encodes the VP13 membrane protein [12]. To gain some indication of the orientation of I5 within the membrane, we labeled intact virions with the anti-V5 serum and a 10 nm-gold particle-conjugated secondary antibody. Virions produced after infection with a virus encoding a wild-type I5 allele lacking the epitope tag served as a control. The vI5V5 virions were readily labeled with the anti-V5 serum (Fig 3C), indicating that the C-terminal epitope tag is accessible on the exterior face of virions. Detection of the A17 protein on the surface of intact virions served as a positive control [37].

Since the epitope was exposed, we tested the same anti-V5 antibody at dilutions ranging from 1:4 to 1:64 for its ability to neutralize the infectivity of vI5V5 virions (incubation at 45°C for 90–360 min); wild-type virions served as a negative control. No neutralization was observed (not shown). Finally, we treated purified vI5V5 virions with chymotrypsin, trypsin or proteinase K and then examined both the virion particles (P) and the supernatant fluid (S) by immunoblot analysis with anti-V5 and anti-D8 sera. The D8 protein was readily cleaved by all three proteases, as has been observed before [38]. No cleavage or loss of the anti-V5 immunoreactive signal was observed after treatment of the virions with trypsin or chymotrypsin. After proteinase K treatment, however, we noted a moderate loss of the I5V5 protein: only ~35% of the immunoreactive signal remained after 30 min of digestion. We interpret these data as evidence that proteinase K can trim the V5-tagged C-terminus of the protein, albeit inefficiently. Because we did not observe any significant change in electrophoretic mobility of the V5-tagged protein, it appears that neither the N-terminus nor the central loop region of the I5V5 protein is readily accessible to protease in intact virions.

### Analysis of an inducible recombinant in which expression of I5V5 is TET-dependent: repression of I5 does not compromise viral replication in BSC40 cells or human diploid fibroblasts

**Figure 4:**
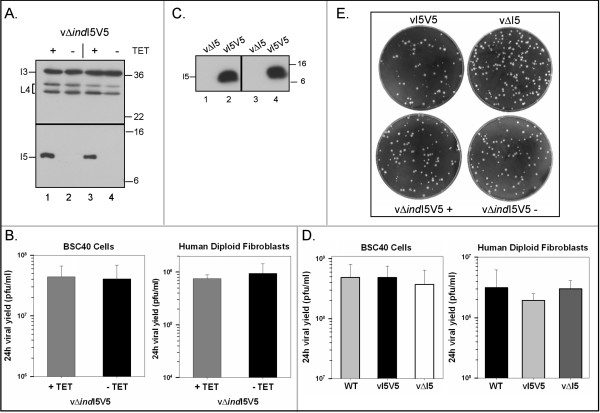
**Repression or deletion of the I5 locus does not have a deleterious effect on virus replication in tissue culture**. *(A) The vΔindI5V5 virus allows tight repression of the I5 protein*. BSC40 cells were infected (MOI 2) with vΔ*ind*I5V5 in the presence (lanes 1,3) or absence (lanes 2,4) of TET. Cells were harvested at 17 hpi and lysates were subjected to immunoblot analysis with the anti-V5 serum (lower panel) or antibodies specific for the intermediate and late viral proteins I3 and L4, respectively (top panel). The molecular masses (in kDa) of protein standards are shown at the right. *(B) Repression of I5 does not affect the viral yield produced in a single infectious cycle in BSC40 cells or primary human fibroblasts*. BSC40 cells or primary human fibroblasts were infected with vΔ*ind*I5V5 (MOI 3) in the presence or absence of TET and harvested at 24 hpi. Viral yield (pfu/ml) was determined by titration on BSC40 cells; experiments were preformed in duplicate and titrated in duplicate. Error bars represent standard deviation. *(C) The vΔI5 virus is deleted for the I5 locus*. Cells were infected with the parental vI5V5 virus (lanes 2,4) or with two isolates of the vΔI5 virus (lanes 1,3) at an MOI 2 and harvested at 18 hpi. Lysates were examined by immunoblot analysis with the anti-V5 antibody. The molecular masses (in kDa) of protein standards are shown at the right. *(D) Deletion of I5 does not affect the viral yield produced in a single infectious cycle in BSC40 cells or primary human fibroblasts*. BSC40 cells or human diploid fibroblasts were infected with wt virus, vI5V5 or vΔI5 (MOI 3) for 24 h, and the viral yield was determined as described for panel B. *(E) Repression or deletion of I5 does not affect viral plaque size*. BSC40 cells were infected with 50–75 PFU of vI5V5, vΔ*ind*I5V5 + TET, vΔ*ind*I5V5 – TET, or vΔI5; plates were fixed and stained with crystal violet at 48 hpi.

To enable a functional characterization of the role of the I5 protein in viral replication, we generated the viral recombinant vΔ*ind*I5V5 (see Fig 1C). To generate this recombinant, we first inserted a bidirectional cassette encoding TetR (a transcriptional repressor) and an inducible copy of the I5V5 gene into the non-essential TK locus of wild-type vaccinia virus, as we have done before for the F10, A13 and I2 genes [19,27,39]. The inducible I5V5 gene is regulated by the endogenous I5 promoter and TetO (the Tet operator). The endogenous I5 locus was then replaced with a NEO cassette that conferred resistance to G418. Our characterization of this virus is shown in Fig 4. Immunoblot analysis showed that, in two isolates of vΔ*ind*I5V5, expression of the I5V5 protein was indeed TET-dependent (panel A, compare lanes 1, 3 with 2, 4). As expected, however, the I3 single-strand DNA binding protein (expressed early, intermediate) and the L4 core protein (expressed late) were expressed under all experimental conditions. Moreover, the relative abundance of both the precursor and processed forms of L4 (upper and lower bands, respectively) was unchanged when I5 was repressed. Since proteolytic processing of the core proteins is coupled to virion morphogenesis, this observation also suggested that repression of I5 might not have any impact on the completion of the viral life cycle [40,41]. Indeed, when we monitored the yield of infectious virus produced at 24 hpi in BSC40 cells (MOI 3), we found that repression of I5 had no impact (panel B, left graph). To assess whether this result would also hold true in primary cells that more closely mimicked replication *in vivo*, we performed the same experiment in primary human foreskin fibroblasts. In this case, too, repression of I5 had no impact on the yield of infectious, cell-associated virus (panel B, right graph).

### Generation of a virus lacking an I5 allele: vΔI5 replicates well in BSC40 cells and human diploid fibroblasts

The results described above strongly suggested that the I5 protein was dispensable in tissue culture; to confirm this result, we generated the vΔI5 virus whose genomic structure is shown in Fig 1C. To make this virus, we began with the vI5V5 virus, and replaced the I5V5 allele with the NEO cassette. This virus was readily isolated, suggesting that the I5L gene was indeed nonessential in tissue culture. To ensure that the genomic structure of the virus was as designed, we performed a number of diagnostic PCR assays to ensure that no I5 allele was present anywhere in the genome and that the NEO cassette had been inserted between the I4L and I6L genes. The results of all of these tests confirmed the correct insertion of the NEO cassette (not shown). The immunoblot in Fig 4C illustrates the loss of I5V5 expression in two isolates of vΔI5. Quantitation of the 24 h viral yield produced in both BSC40 cells and human diploid fibroblasts in shown in Fig 4D: comparable results were obtained with wild-type virus, vI5V5, and vΔI5. We also performed 48 h plaque assays with vI5V5, vΔindI5V5 + and - TET, and vΔI5 on BSC40 cells, and observed that the plaques formed by each of these viruses were indistinguishable in size (4E). Finally, we compared the thermolability of vI5V5 and vΔI5 virions by incubating inocula at 45°C for 90 to 360 min and then performing plaque assays; the absence of I5 had no impact on virus stability in this assay (not shown). Cumulatively, our data indicate that I5 is dispensable for replication in both BSC40 cells and human diploid fibroblasts.

Although the I5 protein is dispensable in tissue culture, its conservation in the genomes of diverse orthopoxviruses suggests that it is likely to have an important role *in vivo*. Indeed, during the preparation of this manuscript, Sood et al reported that I5 does make a significant contribution to the pathogenesis of vaccinia virus in murine models of infection [42]. Because the protein does not show any preferred localization to an intracellular organelle, but is present in developing and mature virions, we propose that it is the encapsidated pool of I5 that would modulate pathogenesis. The protein is highly hydrophobic in nature, and we know that both termini of the protein are likely to be exposed on the virion surface. I5 might play a role in enabling vaccinia virus to infect certain cell types *in vivo*, it might stabilize the virus *in vivo*, or it might bind to cellular ligands in a manner that facilitates infection. Further study of how this small and highly conserved constituent of the virion membrane contributes to pathogenesis will certainly be of interest.

## Conclusion

The 78 aa vaccinia-encoded I5 protein is highly hydrophobic and expressed at late times after infection. I5 is found associated with intermediates in virion morphogenesis as well as mature virions. The C' terminus of I5 is exposed on the surface of intact virions. Repression of I5 expression has no apparent impact on the yield of infectious virus in either BSC40 cells or human diploid fibroblasts; the ability to isolate a virus deleted for the I5L gene supports the conclusion that it is dispensable for replication in tissue culture.

## Competing interests

The authors declare that they have no competing interests.

## Authors' contributions

PT performed the immunoEM studies, contributed to the generation of recombinant viruses, and was responsible for the final version of the manuscript. JN and ES generated the vI5V5 virus and analyzed the expression, post-translational modification and membrane association of the I5V5 protein. BU generated the vΔ*ind*I5V5 and vΔI5 viruses and characterized their replication in culture. BU carried out immunofluorescence assays. BU, JN and ES all participated in drafting of the manuscript.
